# Morbidity and Mortality Predictors of Acute Respiratory Failure in Acute Pancreatitis: A Cohort Study Conducted in Vietnam

**DOI:** 10.1002/jgh3.70136

**Published:** 2025-03-24

**Authors:** Qui Huu Nguyen, Dung Thi My Vo, Thong Duy Vo

**Affiliations:** ^1^ Department of Internal Medicine, Faculty of Medicine University of Medicine and Pharmacy at Ho Chi Minh City Ho Chi Minh city Vietnam; ^2^ Department of Gastroenterology University Medical Center Ho Chi Minh City Ho Chi Minh City Vietnam

**Keywords:** acute pancreatitis, acute respiratory failure, BISAP, SIRS

## Abstract

**Background and Aim:**

Acute pancreatitis (AP) can result in severe complications, with acute respiratory failure (ARF) being among the most critical. Research on ARF in AP remains limited. This study aims to investigate the occurrence, outcomes, and predictors of ARF in AP patients at Cho Ray Hospital, a tertiary care center in Vietnam.

**Method:**

A prospective cohort study was conducted with 230 AP patients at a national hospital in Ho Chi Minh City, Vietnam. Patients were divided into ARF and non‐ARF groups, and clinical characteristics were compared. Key outcomes included invasive mechanical ventilation, in‐hospital mortality, and length of hospital stay.

**Results:**

ARF developed in 26.1% of patients, with a mortality rate of 25.0% in the ARF group versus 1.2% in the non‐ARF group. Mechanical ventilation was required in 48.3% of ARF patients. Significant predictors of ARF were abnormal body mass index (BMI) (*p* = 0.021), prolonged systemic inflammatory response syndrome (SIRS) (*p* < 0.001), modified computed tomography severity index (mCTSI) (*p* = 0.041), and a high bedside index for severity in acute pancreatitis (BISAP) score (*p* < 0.001). BISAP scores ≥ 2 had a sensitivity of 90.0%, specificity of 73.5%, and AUC of 0.878 (95% CI 0.829–0.921) for predicting ARF. Predictors of mortality in ARF patients included cardiovascular failure (HR 15.83, *p* = 0.001), prolonged SIRS (HR 4.76, *p* = 0.038), and high BISAP scores (HR 3.41, *p* = 0.015).

**Conclusion:**

ARF significantly worsens outcomes in AP patients. Early identification of key predictors, like abnormal BMI, prolonged SIRS, mCTSI, and BISAP scores, could improve interventions and patient prognosis.

**Abbreviations**
AP: acute pancreatitis
ARF: acute respiratory failure
AUROC: area under the receiver operating characteristic
BISAP: bedside index for severity in acute pancreatitis
CTSI: computed tomography severity index
HR: hazard ratio
mCTSI: modified computed tomography severity index
NPV: negative predictive value
OR: odd ratio
PPV: positive predictive value
ROC: receiver operating characteristics
Sens: sensitivity
Spec: specificity
WBC: white blood cells

## 1 Introduction

The burden of acute pancreatitis (AP) is increasing globally, both in human and financial terms. In the United States, the incidence rate varies from 4.9 to 73.4 per 100 000 people annually, and annual treatment costs exceed $2.5 billion [1]. A systematic overview from 1990 to 2019 across 204 countries ranked AP sixth in mortality and DALYs among common digestive diseases [2]. Although most cases of acute pancreatitis are mild, with patients improving quickly through fluid resuscitation, pain relief, and early oral feeding, up to 20%–30% of patients suffer from severe acute pancreatitis, with mortality rates in this group reaching up to 30% [3].

Organ failure plays a critical role in the pathophysiology and prognosis of AP. Severe AP is defined when organ failure persists for more than 48 h [4]. Persistent organ failure is associated with mortality in the first week as well as local complications in AP [5]. Acute respiratory failure (ARF) is the most common organ failure in AP [6], with approximately one‐third of patients experiencing acute lung injury (ALI) and acute respiratory distress syndrome (ARDS) [7]. The inflammatory cascade in AP not only affects the pancreas but also other organs, particularly the lungs. The release of pro‐inflammatory cytokines such as TNF‐α, IL‐1β, and IL‐6 leads to endothelial activation and increased vascular permeability, which are central to the development of ALI and ARDS [8]. Notably, the presence of ARF significantly worsens the prognosis of AP, with mortality rates in patients with ARF reported to be as high as 26.5%–80%, compared to less than 5% in those without respiratory complications [6, 9]. Previous research has identified several potential predictors of ARF in AP patients, including sepsis, pleural effusion, pneumonia, and cardiogenic shock [9]. Additionally, factors predicting mortality in these patients are mechanical ventilation, age more than 65 years, sepsis and cancer [9]. These findings highlight the potential utility of these predictors in clinical practice, yet their applicability in different settings remains to be fully explored.

Despite the clinical importance of ARF in AP, comprehensive research on this complication is limited, particularly across diverse healthcare settings. Most studies focus on Western populations, with sparse data from Asian countries, including Vietnam, where epidemiology and clinical characteristics can differ significantly. To address this gap, our study investigates the occurrence, outcomes, and predictors of ARF in AP patients at Cho Ray Hospital, a tertiary care center in Ho Chi Minh City.

## 2 Method

### 2.1 Study Design and Setting

A prospective cohort study was conducted on patients diagnosed with AP admitted to the Department of Gastroenterology of Cho Ray Hospital in Ho Chi Minh City, Vietnam, from November 2023 to June 2024. The inclusion criteria were patients 18 years or older who met the diagnostic criteria for AP according to the Atlanta 2012 revised classification and who agreed to participate in the study. Exclusion criteria comprised patients with insufficient data, chronic pancreatitis, pregnancy, severe pre‐existing conditions such as heart failure, uncontrolled asthma, chronic obstructive pulmonary disease (COPD), Child‐Pugh class B–C cirrhosis, stage 4–5 chronic kidney disease, active cancer, or those not agreeing to participate in the study.

The sample size was calculated from a study by Gajendran et al. [9], showing a 5.4% rate of ARF in AP patients. We estimated the minimum sample size to be 220 based on an absolute precision of 3% with a 95% confidence interval (95% CI) and a 5% level of significance.

### 2.2 Data Collection

We collected data on patients, including age and sex, etiology of AP, body mass index (BMI), signs and symptoms, vital signs at admission, and laboratory data such as leukocyte, hematocrit, platelet, amylase, lipase, triglyceride, C‐reactive protein (CRP), blood urea nitrogen (BUN), creatinine, transaminases, total bilirubin, and total serum calcium. All laboratory results were based on the earliest tests performed after hospital admission, except for CRP, which was measured 48 h after symptom onset. We also documented radiology data on the extent of pancreatic necrosis, Balthazar grade, computed tomography (CT) severity index (CTSI), modified CTSI (mCTSI), and pleural effusion. CT imaging characteristics were derived from scans taken at the time of admission.

The diagnosis of AP is established when at least two of the following three criteria from the revised Atlanta classification 2012 are met: (1) abdominal pain consistent with AP (acute onset of persistent, severe, epigastric pain often radiating to the back); (2) serum amylase or lipase levels elevated to at least three times the upper limit of normal; (3) imaging findings consistent with AP (abdominal ultrasound, computed tomography (CT), or magnetic resonance imaging) [4]. The severity of AP was assessed and classified into three levels: (i) Mild AP is defined as the absence of organ failure and local or systemic complications; (ii) Moderately severe AP is characterized by the presence of transient organ failure or local or systemic complications in the absence of persistent organ failure; (iii) Severe AP (SAP) is characterized by persistent organ failure (lasting more than 48 h).

The diagnosis of ARF was based on the clinical assessment recorded in the medical records by the attending physicians. Patients were followed up until either discharge or the occurrence of mortality.

The bedside index for severity in acute pancreatitis (BISAP) was assessed for predictive abilities. The BISAP score consists of five distinct factors, with each factor contributing one point to the total score. The factors include a BUN level exceeding 25 mg/dL, impaired mental status indicated by a Glasgow Coma Scale (GCS) score below 15, the presence of systemic inflammatory response syndrome (SIRS), age over 60, and the presence of pleural effusion on imaging. The score was recorded at admission and completed within 24 h. A high BISAP score means the score is equal to or greater than 3 [10].

**FIGURE 1:**
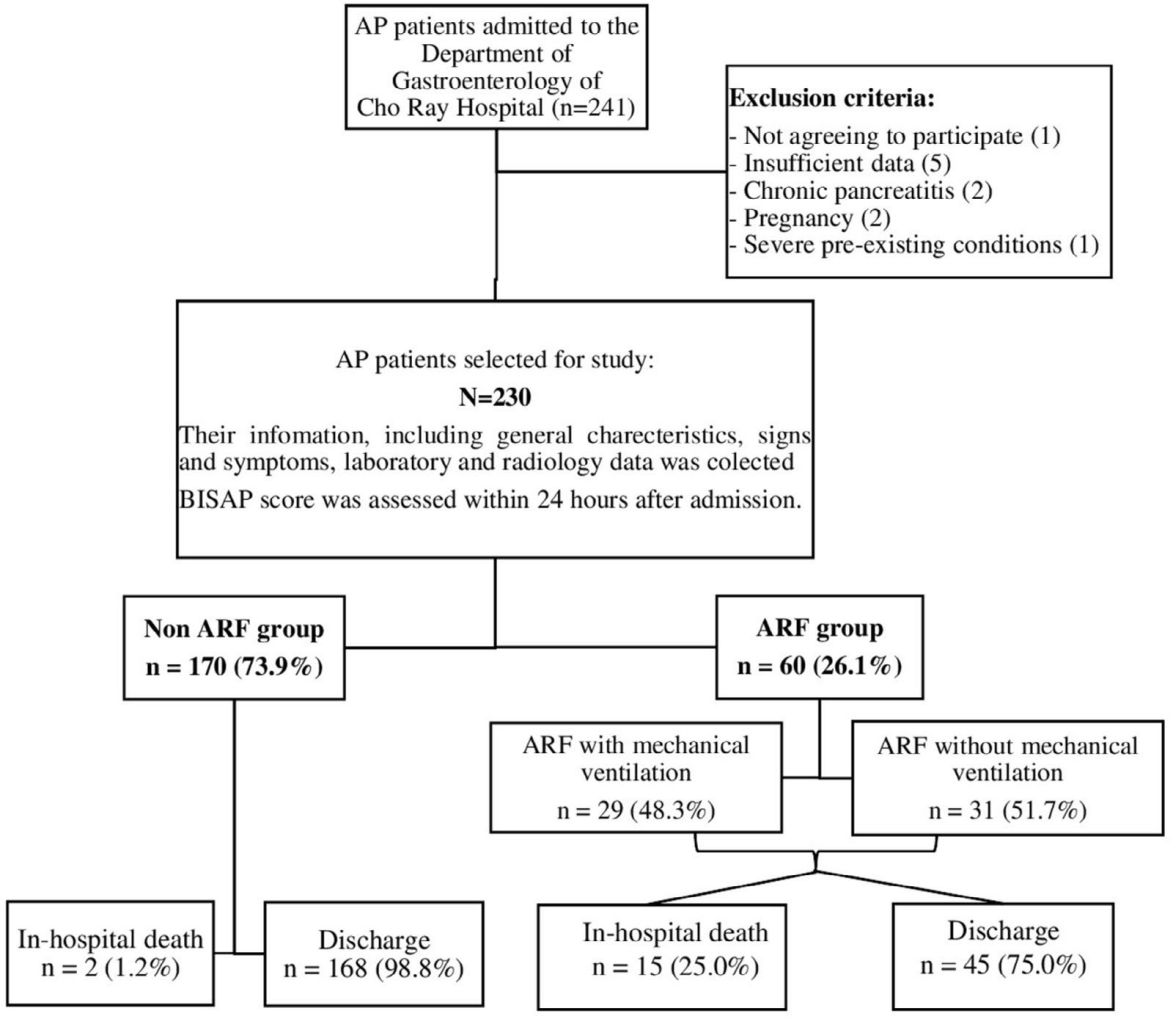
Flowchart of the study (AP, acute pancreatitis; ARF, acute respiratory failure).

The flowchart of the study is illustrated in Figure 1.

### 2.3 Ethics Statement

The study protocol adhered to the ethical principles outlined in the Declaration of Helsinki. The study was approved by the Ethics Committee for Biomedical Research of the University of Medicine and Pharmacy at Ho Chi Minh City (Approval No. 731/HDDD‐DHYD, signed on August 15, 2023). In accordance with national legislation and institutional requirements, written informed consent was not required.

### 2.4 Statistical Analysis

The study utilized Epidata 4.6 for data entry, along with Stata 16.0 for analysis. Descriptive statistics were used for continuous and categorical variables, with comparisons conducted using appropriate tests such as chi‐square, Fisher's exact test, *t*‐test, or Mann–Whitney test. Multivariate regression analysis explored the impact of factors on variables. ROC curves assessed the predictive ability of the BISAP scores for ARF and mortality. To determine the area under the curve (AUC) and the 95% confidence intervals, we utilized bootstrapping statistics for a robust estimation. Statistical significance was determined at *p* < 0.05.

## 3 Result

The study included 230 patients with a mean age of 47.3 ± 15.9 years, of whom 143 (62.2%) were males. Alcohol was the leading cause of AP, comprising 27.8% of cases. Detailed information on patient demographics, clinical characteristics, laboratory, and radiology data is provided in Table 1.

**TABLE 1:** Demographics, clinical characteristics, laboratory data, BISAP score, and outcome of the study population.

Characteristic	Total (*n* = 230)	Non ARF (*n* = 170)	ARF (*n* = 60)	*p*
Age (years), mean (±SD)	47.3 (±15.9)	46.8 (±15.8)	48.8 (±16.3)	0.399
Age categories, *n* (%)				
18–35	56 (24.4)	43 (25.3)	13 (21.7)	0.574
36–50	92 (40.0)	70 (41.2)	22 (36.7)	0.540
51–65	48 (20.9)	32 (18.8)	16 (26.7)	0.199
> 65	34 (14.8)	25 (14.7)	9 (15.0)	0.956
Male gender, *n* (%)	143 (62.2)	104 (61.2)	39 (65.0)	0.600
Etiology, *n* (%)				
Gallstones	57 (24.8)	47 (27.7)	10 (16.7)	0.090
Alcohol	64 (27.8)	46 (27.1)	18 (30.0)	0.662
Hypertriglyceridemia	56 (24.4)	42 (24.7)	14 (23.3)	0.831
Alcohol + hypertriglyceridemia	32 (13.9)	19 (11.2)	13 (21.7)	**0.044**
Other	21 (9.1)	16 (9.4)	5 (8.3)	0.803
History, *n* (%)				
Hypertension	53 (23.0)	30 (17.7)	23 (38.3)	**0.001**
Diabetes	67 (27.0)	45 (26.5)	17 (28.3)	0.780
Smoking	120 (52.2)	85 (50.0)	35 (58.3)	0.267
Using alcohol	123 (53.5)	88 (51.8)	35 (58.3)	0.380
BMI, mean (± SD)	23.4 (±3.6)	23.2 (±3.5)	24.1 (±4.0)	0.104
Normal BMI (18.5–22.9 kg/m^2^), *n* (%)	90 (39.1)	74 (43.5)	16 (26.7)	**0.021**
Abnormal BMI, *n* (%)	140 (60.9)	96 (56.5)	44 (73.3)	**0.021**
BMI Asia‐Pacific classification, *n* (%)				
< 18.5	18 (7.8)	12 (7.1)	6 (10.0)	0.316
18.5–22.9	90 (39.1)	74 (43.5)	16 (26.7)	**0.021**
23–24.9	50 (21.7)	36 (22.2)	14 (23.3)	0.728
≥ 25	72 (31.3)	48 (28.2)	24 (40.0)	0.090
Rebound tenderness/guarding, *n* (%)	17 (7.4)	9 (5.3)	8 (13.3)	**0.044**
Impaired mental status, *n* (%)	9 (3.9)	0 (0.0)	9 (15.0)	**< 0.001**
Laboratory data				
Amylase/lipase level ≥ 3 ULN, *n* (%)	197 (85.7)	142 (83.5)	55 (91.7)	0.122
WBC (G/L)	13.7 (± 5.8)	13.2 (±5.0)	15.0 (±7.6)	**0.043**
Hct (%)	39.5 (± 6.9)	38.4 (±6.1)	42.0 (±8.2)	**0.002**
PLT (G/L)	225 (168–280)	234 (179–284)	190 (132–263)	0.303
Total Calcium (mmol/L)	1.9 (± 0.3)	2.0 (±0.3)	1.7 (±0.3)	**< 0.001**
BUN (mg/dL)	9.0 (13.0–19.0)	12.0 (8.0–16.0)	18.5 (13.0–28.5)	**0.014**
Creatinine (mg/dL)	0.8 (0.7–1.1)	0.8 (0.6–1.0)	1.1 (0.8–1.5)	**0.011**
CRP (mg/dL)	164.6 (90.6–235.5)	146.1 (80.0–215.0)	229.8 (165.2–253.0)	**< 0.001**
Radiology data, *n* (%)				
Balthazar grade E	193 (83.9)	101 (59.4)	46 (76.7)	**0.017**
CTSI ≥ 4	155 (67.4)	107 (62.9)	48 (80.0)	**0.015**
mCTSI ≥ 4	204 (88.7)	146 (85.9)	58 (96.7)	**0.023**
Pancreatic necrosis	77 (33.5)	48 (28.2)	29 (48.3)	**0.005**
Pleural effusion	95 (41.3)	54 (31.8)	41 (68.3)	**< 0.001**
Other organ failure, *n* (%)				
Renal	20 (8.7)	4 (2.4)	16 (26.7)	**< 0.001**
Cardiovascular	7 (3.0)	0 (0.0)	7 (11.7)	**< 0.001**
Renal + Cardiovascular	1 (0.4)	0 (0.0)	1 (1.7)	0.261
Outcome				
Mechanical ventilation, *n* (%)	31 (13.5)	2 (1.2)	29 (48.3)	**< 0.001**
In‐hospital death, *n* (%)	17 (7.4)	2 (1.2)	15 (25.0)	**< 0.001**
Length of stay (days)	6 (5–8)	6 (4–7)	9 (6–12)	**< 0.001**
Predicting tool				
SIRS at admission, *n* (%)	111 (48.3)	60 (35.3)	51 (85.0)	**< 0.001**
Prolonged SIRS, *n* (%)	61 (26.5)	14 (8.2)	47 (78.3)	**< 0.001**
BISAP ≥ 3, *n* (%)	28 (12.2)	3 (1.8)	25 (41.7)	**< 0.001**

*Note:* The bold values indicate statistically significant differences (*p* < 0.05).

ARF occurred in 26.1% of the study population, with 48.3% of these patients requiring mechanical ventilation. As presented in Table 1, no age or gender differences were noted between the ARF and non‐ARF groups. ARF was more common in patients with alcohol and hypertriglyceridemia‐induced pancreatitis (*p* = 0.044) and hypertension (*p* = 0.001), while other histories such as diabetes, alcohol consumption, and smoking did not show any association. Furthermore, normal Asia‐Pacific classification BMI individuals had lower ARF rates (*p* = 0.021). The ARF group had more rebound tenderness/guarding (*p* = 0.044) and altered mental status (*p* < 0.001).

In addition, the ARF group had significantly higher mean/median values in several laboratory tests compared to the control group, including white blood cells (WBC), hematocrit, calcium, BUN, creatinine, and CRP (*p* < 0.05). Radiology data revealed that the Balthazar grade, CTSI, mCTSI score, or the rate of pancreatic necrosis are significantly higher in the ARF group (*p* < 0.05). A significantly greater proportion of patients in the ARF group experienced other organ failures: 26.7% had isolated renal failure and 11.7% had isolated cardiovascular failure (*p* < 0.001).

The BISAP score was higher in the ARF group. Among ARF patients, 41.7% had a high BISAP score (≥ 3), compared to only 1.8% in the non‐ARF group (p < 0.001). The incidence of SIRS at admission and prolonged SIRS was markedly higher in the ARF group than in the non‐ARF group (85.0% vs. 35.3%; 78.3% vs. 8.2%, *p* < 0.001).

The in‐hospital mortality rates of the study population, ARF group, and non‐ARF group were 7.4%, 25.0%, and 1.2%, respectively. The median hospital stay of the ARF group was significantly longer compared to the non‐ARF group (6.0 days vs. 9.0 days, p < 0.001).

A multivariate logistic regression analysis identified significant predictors of acute respiratory failure in AP patients: abnormal BMI (OR 11.46, 95% CI 1.21–108.91, *p* = 0.034), mCTSI score (OR 0.08, 95% CI 0.01–0.90, *p* = 0.041), BISAP score (OR 69.06, 95% CI 4.83–987.54, *p* = 0.002), and prolonged SIRS (OR 492.49, 95% CI 13.59–17849.22, *p* < 0.01) (Table 2).

**TABLE 2:** Multivariate regression analysis of factors predicting ARF in AP patients.

Variable	OR	95% CI	*p*
Hypertension	0.58	0.07–4.64	0.604
Abnormal BMI	11.46	1.21–108.91	**0.034**
Rebound tenderness/guarding	17.34	0.11–2786.98	0.271
WBC	0.99	0.85–1.16	0.943
Hematocrit	1.14	0.96–1.35	0.141
Total Calcium	0.94	0.04–23.84	0.969
BUN	0.90	0.78–1.04	0.157
Creatinine	0.84	0.13–5.33	0.853
CRP	1.00	0.98–1.01	0.737
Balthazar grade	1.65	0.27–9.97	0.585
CTSI	3.74	0.78–17.95	0.100
mCTSI	0.08	0.01–0.90	**0.041**
Pancreatic necrosis	30.01	0.11–8386.36	0.237
Pleural effusion	16.21	0.15–1702.10	0.241
SIRS at admission	0.04	0.00–1.55	0.086
Prolonged SIRS	40.29	17.70–91.68	**< 0.001**
BISAP score	7.89	4.30–14.48	**< 0.001**

*Note:* The bold values indicate statistically significant differences (*p* < 0.05).

**FIGURE 2:**
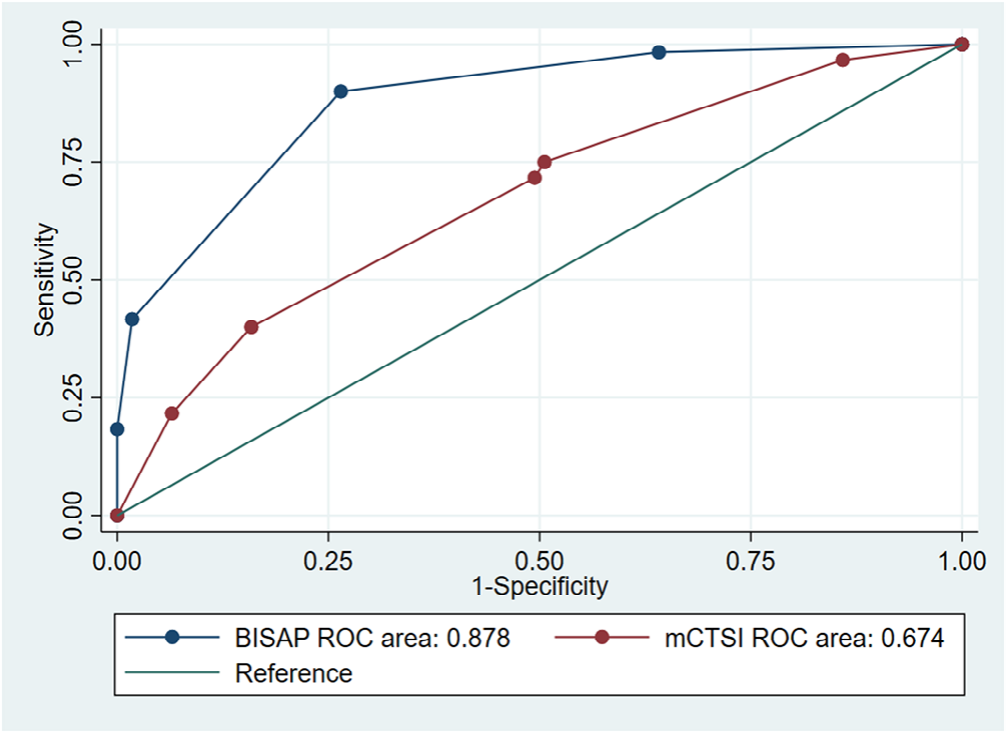
ROC curve of BISAP score and mCTSI in predicting acute respiratory failure in patients with acute pancreatitis (BISAP, bedside index for severity in acute pancreatitis; ROC, receiver operating characteristic; mCTSI, modified computed tomography severity index).

The area under the ROC curve (AUC) values for BISAP and mCTSI for predicting ARF were 0.878 (95% CI 0.829–0.921) and 0.674 (95% CI 0.564–0.748) (Figure 2). The optimal cutoff threshold for the BISAP score in predicting ARF was determined to be 2 points, yielding the highest Youden index, achieving a sensitivity of 90.0%, specificity of 73.5%, positive predictive value of 54.55, and negative predictive value of 95.4%. At an optimal cut‐off of 5, the mCTSI score had a sensitivity of 75.0%, specificity of 49.4%, positive predictive value of 34.4%, and negative predictive value of 84.8% for predicting ARF (Table 3).

**TABLE 3:** Evaluation value of scales in predicting acute respiratory failure in patients with acute pancreatitis.

Scoring system	Cut‐off point	AUC (95% CI)	Sens (%)	Spec (%)	PPV (%)	NPV (%)	*p*
BISAP	≥ 2	0.878 (0.829–0.921)	90.0	73.5	54.5	95.4	**< 0.001**
mCTSI	≥ 5	0.674 (0.564–0.748)	75.0	49.4	34.4	84.8	**< 0.001**

*Note:* The bold values indicate statistically significant differences (*p* < 0.05).

Abbreviations: AUC, area under the curve; BISAP, bedside index for severity in acute pancreatitis; CI, confident interval; mCTSI, modified computed tomography severity index; NPV, negative predictive value; PPV, positive predictive value; ROC, receiver operating characteristics; Sens, sensitivity; Spec, specificity.

In predicting mortality among the ARF group, multivariate Cox regression analysis identified cardiovascular failure (HR 15.83, 95% CI 3.00–83.65), prolonged SIRS (HR 4.76, 95% CI 1.09–20.74), and BISAP score (HR 3.41, 95% CI 1.27–9.15) as significant predictors (Table 4). Specifically, the BISAP score demonstrated a predictive value with an AUROC of 0.705 (95% CI 0.553–0.857). The optimal cutoff threshold for mortality was determined to be 4 points, achieving a sensitivity of 46.7%, specificity of 91.1%, positive predictive value of 63.6%, and negative predictive value of 86.7% (Table 5).

**TABLE 4:** Multivariate analysis for predictors of mortality in AP patients with ARF.

Variable	HR	95% CI	*p*
Impaired mental status	1.03	0.20–5.33	0.976
Cardiovascular failure	15.83	3.00–83.65	**0.001**
Renal failure	4.27	1.09–20.74	0.102
mCTSI	1.07	0.79–1.44	0.673
Prolonged SIRS	4.76	1.09–20.74	**0.038**
BISAP score	3.41	1.27–9.15	**0.015**

*Note:* The bold values indicate statistically significant differences (*p* < 0.05).

**TABLE 5:** Value of BISAP score in predicting mortality in AP patients with ARF.

Scoring system	Cut‐off point	AUC (95% CI)	Sens (%)	Spec (%)	PPV (%)	NPV (%)	*p*
BISAP	≥ 4	0.705 (0.553–0.857)	46.7	91.1	63.6	86.7	**< 0.001**

*Note:* The bold values indicate statistically significant differences (*p* < 0.05).

Abbreviations: AUC, area under the curve; BISAP, bedside index for severity in acute pancreatitis; NPV, negative predictive value; PPV, positive predictive value; ROC, receiver operating characteristics; Sens, sensitivity; Spec, specificity.

## 4 Discussion

Our study represents the first investigation into acute respiratory failure in acute pancreatitis patients in Vietnam. The study revealed a notable incidence of ARF in AP patients, with 26.1% of the cohort experiencing this complication. This rate is consistent with previous studies that reported approximately 30% of patients will develop acute lung injury (ALI) and acute respiratory distress syndrome (ARDS) [7, 11]. Compared to the lower rate of 5.4% reported by Gajendran et al. (2021) [9], our higher incidence may be due to the tertiary hospital setting with more severe cases. Patients who developed ARF had significantly higher rates of concurrent alcohol and hypertriglyceridemia‐induced pancreatitis (21.7% vs. 11.2%, *p* < 0.05), hypertension (38.3% vs. 17.7%, *p* = 0.001), and abnormal BMI (73.3% vs. 56.5%, p < 0.05). This group also exhibited more severe clinical manifestations, including rebound tenderness/guarding and impaired mental status at admission. These findings underscore the importance of vigilant monitoring for these factors upon admission of AP patients. In multicenter studies conducted in China, the incidence of ARDS in patients with acute pancreatitis ranged from 6.55% to 16.6% [12, 13, 14]. These studies also revealed that AP patients with hypertriglyceridemia had a significantly higher incidence of ARDS [12, 13]. Differences in the causes of AP, particularly the higher prevalence of hypertriglyceridemia‐induced AP, may explain why Asian countries have a higher proportion of patients with AP complicated by ARF compared to Western countries.

Many previous studies noted that lung injury in AP is primarily mediated by the release of cytotoxic and vasoactive substances leading to increased pulmonary microvascular permeability and decreased lung compliance [7, 8, 9]. In our study, prolonged SIRS was identified as the leading predictor of ARF in AP patients, with an OR of 40.29 (95% CI 17.70–91.68). The patients with ARF also had significantly higher laboratory markers of inflammation, such as elevated WBC and increased CRP levels. Our results were similar to those of studies conducted in China, showing that patients who developed ARDS had elevated WBC and increased heart rates within the first 24 h [12, 13]. These findings support the notion that a severe systemic inflammatory response is a major driver of ARF in AP. Additionally, radiological findings such as higher Balthazar grade, computed tomography severity index (CTSI), modified CTSI (mCTSI), and presence of pancreatic necrosis were more prevalent in the ARF group. These results suggest that these parameters could serve as early indicators for the development of ARF in AP patients.

The in‐hospital mortality rate for patients with ARF was significantly higher than for those without ARF (25.0% vs. 1.2%, *p* < 0.001). 48.3% of patients in the ARF group required mechanical ventilation. Additionally, the length of hospital stay was longer for ARF patients (9.0 days vs. 6.0 days, p < 0.001), indicating a more severe disease course and higher resource utilization. In our study, AP patients with ARF had a mortality rate similar to that found in Gajendran's study (25% vs. 26.5%) [9]. However, we observed a lower incidence of mechanical ventilation (48.3% vs. 66%). This difference could be attributed to variations in diagnostic criteria for respiratory failure across studies. Notably, our study did not document non‐invasive ventilation methods. Some research reports that non‐invasive ventilation may reduce the need for intubation in AP patients with lung injury [15, 16].

Cardiovascular failure emerged as the leading predictor of mortality in the ARF group (HR 15.83, 95% CI 3.00–83.65, *p* = 0.015). Research conducted by Machicado et al. (2020) has demonstrated that patients with AP who suffer from respiratory failure, circulatory failure, or a combination of both have a significantly higher mortality rate compared to those with other organs dysfunction [6]. The presence of prolonged SIRS was another strong predictor of mortality (HR 4.76, 95% CI 1.09–20.74, *p* = 0.038). This underscores the impact of sustained inflammation on patient outcomes.

In our study, a BISAP score of ≥ 2 predicted ARF with a sensitivity of 90.0% and specificity of 73.5%, and an AUC of 0.878 (95% CI 0.829–0.921). For mortality prediction among ARF patients, a BISAP score of ≥ 4 was identified, achieving a sensitivity of 46.7%, specificity of 91.1%, positive predictive value (PPV) of 63.6%, and negative predictive value (NPV) of 86.7%, with an AUC of 0.705 (95% CI 0.553–0.857). These results are consistent with the study by Yun‐Long Li et al. in China, in which the AUC of BISAP for predicting ARDS was 0.871 (95% CI 0.827–0.916) [13]. Our study focused on predicting mortality specifically in the subset of AP patients with ARF, finding an optimal BISAP score cut‐off of ≥ 4 for mortality. In contrast, Wu et al. identified a BISAP score cut‐off of 3 for predicting mortality in a general AP population, with a sensitivity of 50% and specificity of 91% [10]. The variation in cut‐off values may be due to differences in patient demographics, clinical outcomes, and statistical methods used between the studies.

## 5 Limitations

Our study had several limitations, including its single‐center design and potential selection bias due to the tertiary care setting. Future research should aim to validate our findings in larger, multicenter cohorts and explore targeted therapies to reduce ARF incidence and improve outcomes.

## 6 Conclusion

Our study highlights the significant burden of acute respiratory failure in acute pancreatitis patients, with an incidence of 26.1% and an associated high in‐hospital mortality of 25.0%. Key predictors for ARF included abnormal BMI, prolonged systemic inflammatory response syndrome, mCTSI score, and BISAP score. Mortality in ARF patients was further predicted by cardiovascular failure, prolonged SIRS, and high BISAP scores. These findings underscore the importance of early identification and intervention in high‐risk AP patients to improve outcomes. Further research is needed to validate these predictors in larger, diverse cohorts.

## Consent

Patient consent was obtained.

## Conflicts of Interest

The authors declare no conflicts of interest.

## Data Availability

The data supporting the findings of this study are available from the corresponding author upon reasonable request.
